# Supplementary material for: Age and vaccine information sources drive vaccine hesitancy: A household survey in Central-Western Brazil
Source: PLoS One. 2026 May 5;21(5):e0348412. doi: 10.1371/journal.pone.0348412 (PMC13143092; doi:10.1371/journal.pone.0348412)
Supplement: S1 Appendix — (PDF) [file pone.0348412.s001.pdf]

S1 Appendix. Sampling strategy and sample size calculation using the WHO two-stage cluster sampling method

The sampling method was conducted according to the cluster sampling proposed by the WHO, in 2018, for studies that aim to estimate vaccination coverage. This method is based on two stages: (1) cluster selection and (2) households selection.

(1) Definition and selection of clusters: assuming that the expected average vaccination coverage in Campo Grande, for all vaccines available in the PNI, is 90%, with a confidence interval around the estimates of 8% (i.e., 90% ± 8% coverage estimate), with an alpha (type I error) of 5%, the effective sample size – based on an assumption of simple random sampling – was n = 101 (Table 1).

Table 1 Reproduction of Table B-1 – Effective sample size (ESS) by expected coverage and desired precision for the 95% confidence interval (CI)

Precision for 95% CI	Expected Coverage										
	**5%**	**10%**	**15%**	**20%**	**25%**	**30 - 70%**	**75%**	**80%**	**85%**	**90%**	**95%**
**±3%**	354	518	663	788	892	1,097	892	788	663	518	354
**±4%**	227	315	394	461	517	622	517	461	394	315	227
**±5%**	162	216	265	306	340	401	340	306	265	216	162
**±6%**	132	160	192	220	242	280	242	220	192	160	132
**±7%**	110	125	147	167	182	207	182	167	147	125	110
**±8%**	93	101	117	131	143	159	143	131	117	**101**	93
**±9%**	81	83	96	106	115	126	115	106	96	83	81
**±10%**	70	70	80	88	95	103	95	88	80	70	70

Source: Annex B-1 of the WHO 2018 manual

To determine the average number of people eligible for the study (individuals aged 12 years or older), a pilot study was conducted. For this, one cluster (census tract) was randomly selected. Considering that on average, in Brazil, each census tract has approximately 300 households, for the pilot study 10% of the households contained in the selected cluster were drawn, which resulted in 31 households. After completing the pilot study, the average number of respondents per cluster, within a 3-hour interval, with a field team of 6 researchers, distributed in pairs, was 10. Assuming that the intracluster correlation is 0.33, the design effect size was set at 3.

Average Respondents per Cluster (m)							
**ICC**	**1**	**5**	**7**	**10**	**15**	**20**	**Description**
**0**	1	1	1	1	1	1	Uniform coverage
**0.042**	1	1.17	1.25	1.38	1.58	1.79	ICC = 1/24; very little variation in coverage
**0.167**	1	1.67	2	2.50	3.33	4.17	ICC = 1/6; conservative choice for SIA surveys
**0.333**	1	2.33	3	4	5.67	7.33	ICC = 1/3; conservative choice for RI surveys
**1**	1	5	7	10	15	20	Some clusters 100% covered; all others 0%

Applying the formula proposed by the WHO, the estimated number of clusters was 30.3, which was rounded to 30 (equation **a**). The selection of clusters was done by simple random sampling without replacement, using the cartographic base of census sectors from the IBGE of 2021. The cluster where the pilot study was conducted was included in the study. Therefore, an additional 29 clusters were sampled afterward. Clusters that primarily contained institutionalized populations (prisons and long-term care facilities, such as nursing homes) were immediately replaced when drawn. Clusters containing large condominiums or gated communities that did not allow the study team entry for data collection after initial contact by the researchers were also replaced (Figure 1).

$$\textbf{(a)}\ \boldsymbol{N}\text{clusters} = \frac{\boldsymbol{ESS}\times\boldsymbol{DEFF}}{\boldsymbol{m}} \rightarrow \frac{\mathbf{101\times3}}{\mathbf{10}} \rightarrow \mathbf{30.3} \rightarrow \mathbf{30} *$$

Table Summary of terms of equations for sampling

**Item**	**Equation term**	**Input**		**Term value**
a	Number of strata	No input		1
b	Effective sample size estimation with desired precision (ESS)	Expected coverage	90%	101
		Precision level	8%	
c	Design effect	m (eligible participants per cluster)	10	3

		ICC – Intracluster correlation coefficient	0.33	
		CV – coefficient of variation	1.05	
d	Number of households to visit to find an eligible participant	No input		1.5
e	Nonresponse	Eligible and not respond		1.05

Source: Adapted from WHO 2018 manual

Figure 1 Selected clusters for the household survey.

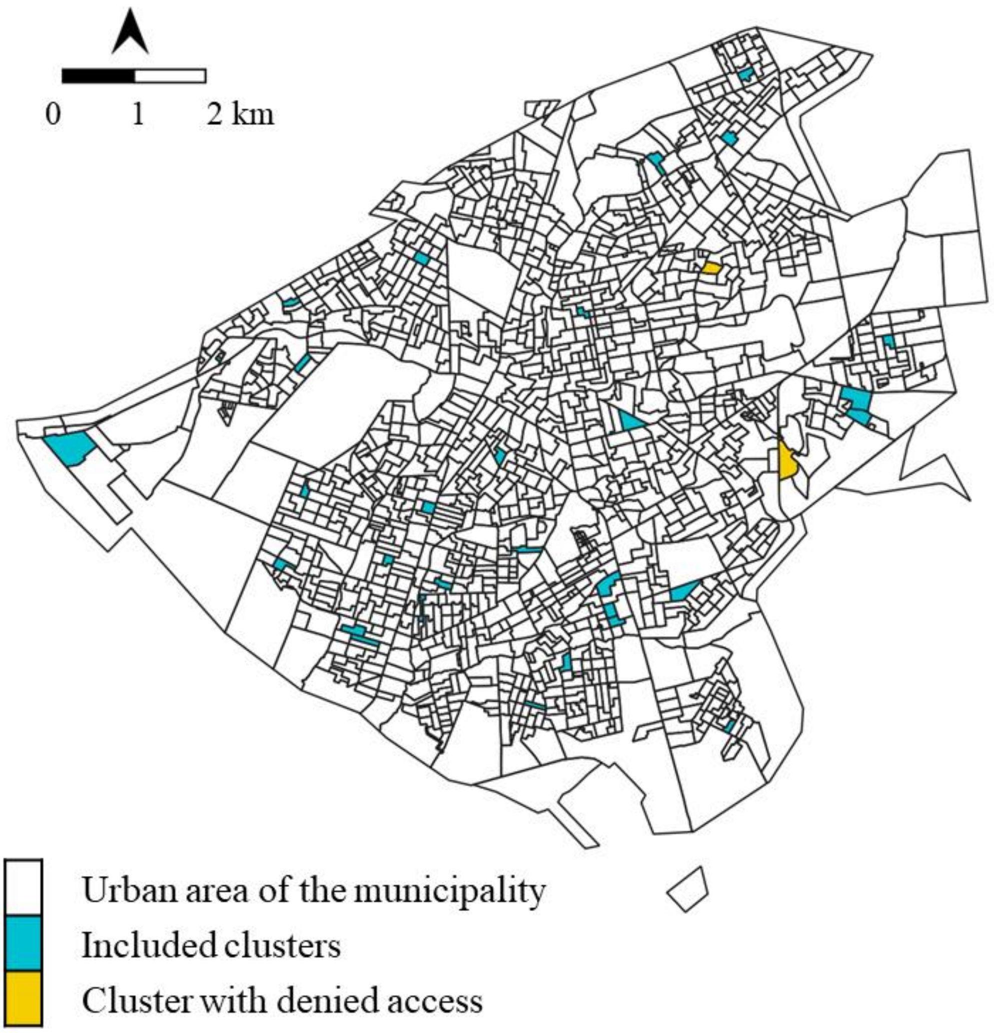

Source: Adapted from IBGE cartographic base of census sectors

(2) The definition of the number and selection of the households was based on the pilot study. The number of residences visited to find an eligible participant averaged 1.5, the inflation factor to account for refusals and non-respondent residences was 1.05, and the average number of respondents per day of data collection was 10. Therefore, the number of households per cluster was defined as 15 (equation **b**).

$$\textbf{(b)}\ \boldsymbol{N}\text{holseholds to visit per cluster} = \boldsymbol{D} \times \boldsymbol{E} \times \boldsymbol{m} \rightarrow \mathbf{1.5} \times \mathbf{1.05} \times \mathbf{10} = \mathbf{15}$$

Those households were selected by random simple probabilistic sampling. The random sampling and spatial allocation of clusters and households were performed using *sf* package from software R 3.4.2.

References

WAGAI, J. N.; RHODA, D.; PRIER, M.; TRIMMER, M. K.; CLARY, C. B.; OTERI, J.; OKPOSEN, B.; ADENIRAN, A.; DANOVARO-HOLLIDAY, C.; CUTTS, F. Implementing WHO guidance on conducting and analysing vaccination coverage cluster surveys: Two examples from Nigeria. **PLoS ONE**, v. 16, n. 2 Febuary, p. 1–24, 2021. Disponível em: <http://dx.doi.org/10.1371/journal.pone.0247415>.

WHO. **World Health Organization Vaccination Coverage Cluster Surveys: Reference ManualWorld Health Organization**2018. Disponível em: <http://www.who.int/immunization/documents/who_ivb_18.09/en/>.

IBGE. **Malha de Setores Censitários**. 2022. Disponível em: https://www.ibge.gov.br/geociencias/organizacao-do-territorio/estrutura-territorial/26565-malhas-de-setores-censitarios-divisoes-intramunicipais.html. .
