# Supplementary material for: Age and vaccine information sources drive vaccine hesitancy: A household survey in Central-Western Brazil
Source: PLoS One. 2026 May 5;21(5):e0348412. doi: 10.1371/journal.pone.0348412 (PMC13143092; doi:10.1371/journal.pone.0348412)
Supplement: S2 Appendix — (PDF) [file pone.0348412.s002.pdf]

S2 Appendix. Analysis of vaccine hesitancy by age groups (20-year intervals): descriptive comparisons and multivariable logistic regression

Table A. Stratified age data according to the occurrence of vaccine hesitancy

	**Total** *(n=518)*	**NHP** *(n=258)*	**HP** *(n=260)*	**OR (95%CI)**	**p-value**
Stratified age					<0.001
12 – 31	124 (23.9%)	54 (20.9%)	70 (26.9%)	Ref.	
32 – 51	174 (33.6%)	70 (27.1%)	104 (40.0%)	1.15 [0.72;1.83]	
52 – 71	179 (34.6%)	108 (41.9%)	71 (27.3%)	0.51 [0.32;0.81]	
72 or more	41 (7.92%)	26 (10.1%)	15 (5.77%)	0.45 [0.21;0.92]	

Table B. The final model for the occurrence of vaccine hesitancy.

**Covariates**	**β Coefficient (SE)**	**aOR (95% CI)**
(Intercept)	2.12 (0.66)	8.38 (2.49; 34.87)
Age (32 – 51 years old)	0.26 (0.24)	1.30 (0.80;2.12)
Age (52 – 71 years old)	− 0.53* (0.24)	0.59 (0.36; 0.95)
Age (72 years old or more)	− 0.58 (0.38)	0.56 (0.26; 1.18)
"Do you believe that vaccines can protect yourself and children from serious diseases?" (Yes)	− 1.46* (0.58)	0.23 (0.06; 0.66)
Main source of information about vaccination (Health care worker/websites or profile of official Health Organizations)	− 0.99* (0.40)	0.37 (0.16; 0.81)
Main source of information about vaccination (social media)	− 0.01 (0.41)	1.00 (0.43; 2.22)
Main source of information about vaccination (Traditional media: online and television journals)	− 0.67 (0.39)	0.51 (0.23; 1.08)

Legend: SE: Standard Error; aOR: adjusted odds ratio; CI: confidence interval
*p-value < 0.05; **p-value < 0.01
AIC: 686.8
Hosmer and Lemeshow goodness of fit (GOF) test: X-squared = 2.4027, df = 7, p-value = 0.9342
