# Supplementary material for: Age and vaccine information sources drive vaccine hesitancy: A household survey in Central-Western Brazil
Source: PLoS One. 2026 May 5;21(5):e0348412. doi: 10.1371/journal.pone.0348412 (PMC13143092; doi:10.1371/journal.pone.0348412)
Supplement: S3 Appendix — (PDF) [file pone.0348412.s003.pdf]

S3 Appendix. Data collection instrument: adapted WHO SAGE questionnaire, additional variables, and 3C model framework for vaccine hesitancy

1. Date of birth:

2. Age:

3. Sex:
( ) Male ( ) Female

4. Color/ethnicity:
a) Yellow
b) White
c) Brown
d) Black
e) Indigenous

5. Education:
a) Illiterate
b) Incomplete primary education
c) Complete primary education
d) Incomplete secondary education
e) Complete secondary education
f) Incomplete higher education
g) Complete higher education

6. What was the last series you completed?
6.1. Schooling in years of study:

7. Current occupation:

8. Approximate family income (Brazilian real or minimum wage):

9. Number of people living in the household (including yourself):

10. Does the residence have access to treated water?
( ) Yes ( ) No

11. Does the residence have access to sanitary sewer?
( ) Yes ( ) No

12. Do you have health insurance?
( ) Yes ( ) No

13. For how long have you been living in Campo Grande?

14. Do you have children or are you a legal guardian of children under your care?
( ) Yes ( ) No
14.1. If "YES", how many of them are under 12 years old?

15. Do you believe that vaccines can protect you and children from serious diseases?
( ) Yes ( ) No

15.1. Has this answer changed since the start of the COVID-19 pandemic?
( ) Yes ( ) No

16. Do you think most of the people you know get all the recommended vaccines?
( ) Yes ( ) No

16.1. Has this answer changed since the start of the COVID-19 pandemic?
( ) Yes ( ) No

17. "The following questions relate to the concept of vaccine hesitancy, which is the delay in accepting or even refusing vaccination regardless of the availability of vaccines in the SUS (Brazilian Public Health System). Hesitation involves personal factors (such as religion, culture, and politics), factors related to trust in the health system and available vaccines (including fear of adverse effects), and the importance of vaccination and the convenience of getting vaccinated (difficulty missing work, not perceiving the need for vaccination, etc.)." So, have you ever felt reluctant or hesitant about getting vaccinated?
( ) Yes ( ) No

17.1. (If the answer is "YES") Did this hesitancy occur before or after the start of the COVID-19 pandemic?
( ) Before ( ) After

18. Have you ever refused a vaccine?
( ) Yes ( ) No

18.1. If the answer is "YES", did this refusal occur before or after the start of the COVID-19 pandemic?
( ) Before ( ) After

19. If the answer is "YES" to questions 17 or 18, indicate which vaccines you have hesitated to get administered:

20. If the answer is "YES" to questions 18 or 19, some of these reasons related to vaccine confidence apply (you may select more than one option):
a. I heard or read negative things about vaccines in the media.
b. I didn't think the vaccine was effective.
c. I didn't think the vaccine was safe and was worried about side effects.
d. Someone else told me the vaccine wasn't safe.
e. I had a bad experience with a healthcare professional who administered the vaccine in a previous vaccination (or a bad experience with the healthcare facility).
f. Someone else told me that they or one of their children had a bad post-vaccination reaction.
g. I had a bad experience or post-vaccination reaction with a previous vaccination.
h. Not applicable.

20.1. Were some of the items marked motivated by the COVID-19 pandemic? If so, indicate which ones:

21. If the answer is "YES" to questions 17 or 18, indicate whether any of these reasons related to the importance and convenience of getting vaccinated are applicable (you may select more than one option):
a. I didn't think it was necessary.
b. I didn't know where to get the vaccine.
c. I didn't know where to get good, reliable information about vaccines.
d. I couldn't go to the health center to get vaccinated because I can't miss work.

e. Not applicable

21.1. Were some of the items marked motivated by the COVID-19 pandemic? If so, indicate which ones:

22. If the answer is "YES" to questions 17 or 18, indicate whether any of these individual reasons apply to your case (you may select more than one option):

a. I'm afraid of needles
b. Religious reasons
c. Political reasons
d. Other beliefs/traditional medicine/alternative medicine
e. Not applicable

22.1. Were some of the items marked motivated by the COVID-19 pandemic? If so, indicate which ones:

23. Have distance, opening hours, travel time to the health unit, waiting time, or even a lack of vaccines ever prevented you from getting vaccinated?

( ) Yes ( ) No

23.1. (If the answer is "YES") Indicate which of these was the main reason for not vaccinating:

a) Distance
b) Opening hours
c) Time required to reach the unit
d) Waiting time
e) Lack of vaccines

24. Are there other reasons or motives in your life that prevent or have prevented you from getting vaccinated on the correct date?

( ) Yes ( ) No

24.1. (If the answer is "YES") Could you please indicate or specify what those reasons are?

24.2. (If the answer is "YES") Did these reasons appear before or after the start of the COVID-19 pandemic?

( ) Before ( ) After

25. Do you believe there is any reason why people should not be vaccinated?

( ) Yes ( ) No

25.1. (If the answer is "YES") Could you please indicate or specify what those reasons are?

25.2. (If the answer is "YES") Did these reasons appear before or after the start of the COVID-19 pandemic?

( ) Before ( ) After

26. Do you believe that some community groups (such as ethnic and/or religious groups) in your community or region have difficulty getting vaccinated?

( ) Yes ( ) No ( ) Doesn't know?

27. (If the answer is "YES" to question 26) Indicate whether any of these reasons apply (you may select more than one option):

a) They choose not to get vaccinated.
b) They don't feel welcome at the health unit.
c) The health units don't reach them (they don't conduct active outreach or home visits).
d) Other. If there is another reason, please specify:

28. Have you ever received or heard negative information about vaccines?

( ) Yes ( ) No

28.1. (If the answer is "YES") Did this information influence your decision to get vaccinated?

( ) Yes ( ) No

28.2. (If the answer is "YES") Was this before or after the start of the COVID-19 pandemic?
( ) Before ( ) After

29. Do leaders in your community or neighborhood (religious leaders, politicians, teachers, healthcare professionals) discourage vaccination?
( ) Yes ( ) No ( ) Doesn't know?

29.1. (If the answer is "YES") Indicate which leaders discourage vaccination in your community:

a) Religious
b) Political
c) Teachers
d) Healthcare professionals
e) Other. If other, please specify:

30. What is the source of information you use most frequently to learn about vaccines? (Check only 1 option)

a) None
b) Social networks (YouTube, Facebook, Twitter, Instagram, WhatsApp, etc.)
c) News websites
d) Websites or social media profiles of official health organizations (SESAU, Ministry of Health, World Health Organization)
e) Friends
f) Neighbors
g) Healthcare professional
h) Television
i) Other. If another source of information, specify:

31. Have you received any guidance from healthcare professionals about the importance of getting vaccinated?
( ) Yes ( ) No ( ) Doesn't know/doesn't remember

32. How would you rate your relationship with the healthcare professionals at your neighborhood's primary healthcare unit?

a) Excellent
b) Good
c) Fair
d) Poor
e) Indifferent
f) I do not frequent any basic health unit

33. How many doses of the COVID-19 vaccine have you received?

a) 1
b) 2
c) 3
d) 4
e) None
